# Artificial intelligence-driven transformations in diabetes care: a comprehensive literature review

**DOI:** 10.1097/MS9.0000000000002369

**Published:** 2024-07-23

**Authors:** Muhammad Iftikhar, Muhammad Saqib, Sardar Noman Qayyum, Rehana Asmat, Hassan Mumtaz, Muhammad Rehan, Irfan Ullah, Iftikhar Ud-din, Samim Noori, Maleeka Khan, Ehtisham Rehman, Zain Ejaz

**Affiliations:** a Khyber Medical College, Peshawar; b Bacha Khan Medical College, Mardan; c Gomal Medical College, Dera Ismail Khan; d Al-Nafees Medical College and Hospital, Islamabad, Pakistan; e BPP University, London, UK; f Nangarhar University, Faculty of Medicine, Nangarhar, Afghanistan

**Keywords:** artificial intelligence, closed-loop systems, and insulin pumps, diabetes mellitus, diabetes research, machine learning, metabolic diseases, self-management apps

## Abstract

Artificial intelligence (AI) has been applied in healthcare for diagnosis, treatments, disease management, and for studying underlying mechanisms and disease complications in diseases like diabetes and metabolic disorders. This review is a comprehensive overview of various applications of AI in the healthcare system for managing diabetes. A literature search was conducted on PubMed to locate studies integrating AI in the diagnosis, treatment, management and prevention of diabetes. As diabetes is now considered a pandemic now so employing AI and machine learning approaches can be applied to limit diabetes in areas with higher prevalence. Machine learning algorithms can visualize big datasets, and make predictions. AI-powered mobile apps and the closed-loop system automated glucose monitoring and insulin delivery can lower the burden on insulin. AI can help identify disease markers and potential risk factors as well. While promising, AI’s integration in the medical field is still challenging due to privacy, data security, bias, and transparency. Overall, AI’s potential can be harnessed for better patient outcomes through personalized treatment.

## Introduction

Highlights

- Machine learning (ML) algorithms such as k-nearest neighbor, support vector machine, naïve Bayes, gradient boosting have been applied to develop classifier systems for diabetes diagnosis with upto 99% accuracies.
- Deep learning (DL) algorithms such as recurrent neural networks, convolutional neural networks, LSTM, etc. have outperformed the conventional ML models in diabetes detection.
- AI can also differentiate between various types of diabetes such as type 1 diabetes mellitus, type 2 diabetes mellitus, gestational diabetes mellitus, etc.
- Novel approaches like tongue appearance and pulse pressure from traditional Chinese medicine combine with ML have demonstrated good results for noninvasive diabetes screening.
- AI-powered mobile apps and wearables can also provide real-time glucose monitoring, personalized feedbacks, and insulin dosage recommendations to diabetes patients.
- AI-based closed-loop pancreatic systems can also automate insulin delivery using insulin pumps, and continuous glucose monitoring.

Artificial intelligence makes machines capable of simulating intelligence and endows machines with human-like abilities such as learning, reasoning, interpreting, decision-making, and problem solving. Nowadays AI is being employed in healthcare setups for a variety of purposes such as disease diagnosis, prediction new onset, personalized treatment, and medical development. AI algorithms can analyze patient data, that is, from diagnostic images and lab results to demographic data. Machine learning, an AI subtype, has demonstrated AI’s potential in detection and management of diabetes and other metabolic disorders. Deep learning, a ML subtype, demonstrated physicians’ level accuracy in interpreting diagnostic images and making diagnoses^1^. Elevated glucose levels are a hallmark of diabetes, a chronic metabolic condition caused by inefficiencies in insulin production or action. The term ‘metabolic diseases’ refers to a set of conditions that impair the body’s capacity to turn food into energy and result in abnormal blood levels of certain substances such as glucose, cholesterol, triglycerides, etc. Approximately 8.4 million people^2^ worldwide had type 1 diabetes in global statistics of 2021. Of these 18% were under 20 years old. There were 500 000 new cases on average^2^ diagnosed globally, with an average onset of 9 years.

Diabetes and metabolic disorders are associated with higher rates of morbidity with expanding public health burden on a global scale^3^. AI algorithms have been developed that can automate screening and help manage diabetes and other metabolic disorders. It presents a possible route of tackling challenges in the treatment of diabetes and metabolic illnesses by leveraging the capabilities of large-scale data analysis and predictive modeling. However, there is a need to critically assess the use of these AI technologies in the management of diabetes and metabolic disorders given the growing interest of AI in healthcare. This review offers a thorough overview of the present body of information about the use of AI in the diagnosis, treatment and management of metabolic illnesses like diabetes etc. as well as discusses the advantages, and drawbacks. Table 1 summarizes the applications of AI in diabetes care.

**Table 1:** Applications of AI in diabetes care

Applications	Description
Diagnosis of diabetes and it’s subtypes	Based on glycemic profile of an individual, AI can diagnose diabetes and can also classify T1DM, T2DM and GDM with physician’s level accuracy
Early prediction of diabetes	Diabetes onset can be predicted by various algorithms trained on electronic health record data especially predicting GDM during early pregnancy
Glycemic control	AI-integrated insulin pumps are utilized in a large number of studies which automate the insulin infusion rates as per the continuous glucose monitoring (CGM)
Glycemic events prediction	Hyperglycemia or hypoglycemia can also be predicted based on CGM data by the AI algorithms. This technique is available for commercial use as well
Novel markers identification	AI models identify various predictors for diabetes such as age, waist, BMI, hypertension, etc.
Diabetes retinopathy prediction	Risks for diabetic retinopathy can also be predicted using clinical dataset, thus help initiating early management
Diabetes complications diagnosis	Serious diabetes complications such as diabetic foot, diabetic retinopathy and patients at risk of rehospitalization can also be identified by various AI/ML approaches used in various studies

## Methods

A systematic literature search was conducted on PubMed, Google Scholar, PLOS One, and Scopus to identify relevant literature published in the last 10 years, that is, from May 2013 to August 2023 using the keywords: ‘artificial intelligence’, ‘machine learning’, ‘diabetes diagnosis’, ‘diabetes management’, ‘diabetes treatment’. We only included original studies that were available in English language. Review articles, conference abstracts, editorials, and studies that were not available in English language were excluded. We screened 36 studies that met our inclusion criteria to use in this comprehensive review.

## AI applications in diabetes diagnosis

**Figure 1:**
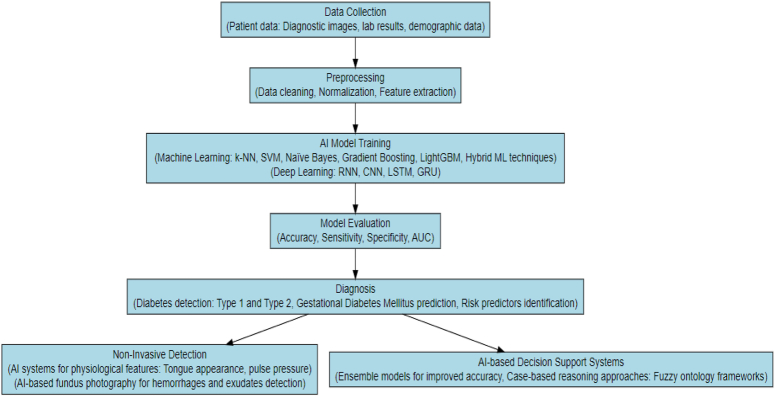
AI applications in diabetes diagnosis.

Diabetes is still diagnosed conventionally via fasting glucose levels and random glucose levels. Smart medicine is a medical model that integrates AI in medicine to assist in diagnosis and management. One of the most fundamental applications of AI in medicine is to automate disease detection and diagnosis with accuracy higher or comparable with physicians. AI-based diabetes diagnostic systems can not only identify diabetes but also classify subtypes of diabetes. As inputs, various AI classifier systems trained on glycemic levels, symptoms such as excessive thirst, frequent urination, and risk factors such as family history, lifestyle, other comorbid conditions, etc., have achieved variable accuracies. Figure 1 underscores the applications of AI in diabetes diagnosis.

### Machine learning for diabetes diagnosis

Machine learning (ML) algorithms make machines imitate humans and learn from the data to make predictions. Various ML algorithms such as k-nearest neighbor (k-NN), support vector machine (SVM), naïve Bayes, Gradient Boosting, etc. have been employed to develop classifier systems for diabetes. The k-NN, SVM, naïve Bayes, and Gradient Boosting are supervised ML algorithms used extensively for classifications, patterns identification, image classification, gene expression analysis, and anomaly detection. SVMs are highly adoptable and can be used for linear and non-linear data^4^.

SVM-based classifier system achieved an accuracy of 87% in a study^4^. In another study, a LightGBM (light gradient boosting machine) based classifier system, which can handle large datasets and used for efficient and accurate predictions and classifications, was used to classify diabetics versus nondiabetics using multimodal input such as age, sex, BMI, blood pressure, cholesterol, insulin, etc. Rufo *et al*.^5^ optimized LightGBM and achieved an accuracy of 98.1% thus optimized LightGBM outperformed all other ML algorithms such as k-NN, SVM, naïve Bayes, etc. with AUC, sensitivity and specificity of 0.98, 99.9, and 96.3%, respectively. A study demonstrated the use of non-linear dynamics analysis such as DFA (detrended fluctuation analysis) and Poincare plot analysis along with machine learning in a diabetes diagnostic model. This ML-based classifier demonstrated an accuracy of 94%^6^. In another study, a hybrid ML technique and metaheuristic algorithms involving k-NN were developed to diagnose diabetes with an accuracy of 91.65% on the PID (Pima Indian Diabetes) dataset^7^. The PID dataset has been developed by National Institute of Diabetes and Digestive and Kidney Disease (NIDDK) and is a well-known dataset for developing ML models. It contains information of 768 women, aged 21 or older, from a population near Phoenix, Arizona, USA. It contains patient features such as pregnancies, plasma glucose levels measured through oral glucose tolerance test, blood pressure, skin thickness, serum insulin, BMI, age, and diabetes pedigree function. The outcome is a binary variable with 1 indicating that patient has diabetes and 0 indicating no diabetes. The diagnostic model^7^ also spotlights the most important risk predictors as blood pressure, blood glucose, and insulin.

### Noninvasive diabetes detection using AI

As medical facilities are not evenly distributed in a country resulting in late diagnosis of chronic diseases such as diabetes. However, an application of ML is the noninvasive detection of diabetes from tongue appearances and pulse pressure evaluation. ML devices can help early diagnosis of diabetes in remote areas lacking medical resources. These physiological features such as tongue appearance and pulse pressure are used in traditional Chinese Medicine for diagnosis. Xiang *et al*.^8^ developed a ML-based classifier to detect diabetes using these physiological features and achieved an accuracy of 85%. They included 165 subjects from 11 medical institutes in Tianjin, China. Fundus photographs were used to detect hemorrhages and exudates while ML devices were used to evaluate tongue appearance and pulse pressure. Xiang *et al*.’s ML-based classifiers outperformed fundus photography with precision, recall, and F1 score of 0.89, 0.67, and 0.76, respectively. This AI diagnostic model can serve as a decision support system in diabetes detection in regions with limited or no medical facilities.

### Deep learning for diabetes diagnosis

Deep learning, a subset of ML, represents a set of complex algorithms that has a neural network structure to mimic the human brain and may not require human intervention for corrections in predictions. DL algorithms include recurrent neural networks (RNN), convolutional neural network (CNN), long short-term memory (LSTM), gated recurrent unit (GRU), etc.

In a standard neural network, all inputs and outputs are independents, but RNNs have introduced the memory to neural networks in which output from previous step functions as input for the next step. It is like reading the book while using the context of previous words to understand the current ones. The RNNs are employed for predictions, text generations, etc. However, simple RNNs struggle with long-term dependencies due to vanishing gradient problem, where the influence of earlier inputs diminishes over time. LSTM addresses this shortcoming of RNNs and have a cell state that runs through entire chain, with the ability to add or remove information regulated by structures known as gates that include forget gate, input gate, and output gate. This architecture allows to learn long-term dependencies. GRU simplifies the LSTM architecture and combines the forget and input gates into a single ‘update gate’, making them faster to train, with similar or better performance than LSTMs^8^.

In a study various DL-based classifier systems were compared, LSTM achieved 97%^4^ accuracy indicative of DL model’s high performance as compared to conventional ML models. To achieve an ideal classifier system, that is, with 100% accuracy, then two DL algorithms (GRU and LSTM) were fused using Dempster–Shafter theory (to deal with conflicts while fusing two models) to achieve an ideal classifier system. However, this ensemble model could achieve 98% accuracy but outperformed the individual ML (87%) and DL (97%) classifier systems. Ensemble models were also developed from ML (k-NN, naïve Bayes, and SVM) and DL (ANN) fusion to classify diabetic versus nondiabetic. This AI classifier system achieved 98.6% accuracy higher than the previous ensemble system^8^.

Neural networks are deep learning algorithms that mimic the human brain and do simulations as physicians. AI systems based on neural networks are highly efficacious in diagnosis and classification of diabetes types and risk predicting. A neural network-based diabetes diagnostic system could classify type 1 and type 2 diabetes in addition with detection with an accuracy of 92% based on blood glucose levels and risk factors^9^. Neural networks such as ANN can also be specified for types of diabetes.

### AI for gestational diabetes mellitus (GDM) prediction

About 2–10% of pregnancies may develop gestational diabetes mellitus (GDM). Neural networks have been found effective in not only predicting GDM bur also helping identify clinical and novel biomarkers for GDM. Wu *et al*.^10^ in a study applied deep neural network-based models for predicting GDM and identifying novel biochemical markers for GDM. This neural network model was trained on 16 819 pregnant women’s electronic data and identified 17 important clinical markers for GDM with an AUC of 0.80. Advanced deep learning techniques and seven variable logistic regression then spotlight seven most important clinical markers and each variable’s contribution. Thus, the study found that a lower BMI ≤17 is associated with GDM and lipoprotein as a novel biomarker for GDM. Shen *et al*.^11^ developed an AI model in a study to identify GDM. This AI model, based on various ML algorithms, achieved higher specificity when compared to human experts. However, sensitivity, accuracy and AUC need further model refinement.

### Case-based reasoning approaches

Case-based reasoning (CBR) is another AI approach to seek solutions for existing problems using prior knowledge and experience from previous cases. A fuzzy ontology-based CBR framework was developed in a study by El-Sappagh *et al*.^12^. A Fuzzy ontology framework can be applied to vague and imprecise data for diagnosis. This CBR-based Fuzzy system was applied to 60 real patient cases with 105 fuzzy data types achieving 97.67% accuracy.

Recently researchers have developed AI, ML, and DL models with the potential to improve the prediction and diagnosis of diabetes. These models can analyze diverse datasets including glucose measurements, medical imaging, and other medical tests to identify patterns not visible to physicians. ANN and SVM have demonstrated the diagnostic accuracy of human experts. The use of AI to automate diabetes screening could be valuable during situations where healthcare resources are strained such as COVID-19 pandemic.

## Applications of AI in diabetes management

**Figure 2:**
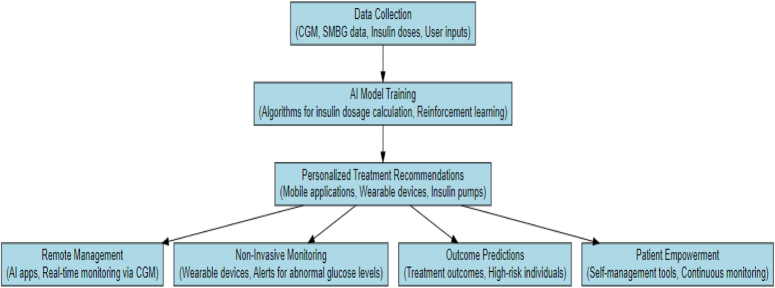
AI applications in diabetes management.

AI is being used more and more in the healthcare industry, particularly in the areas of early diagnosis, management, and therapy. When the body is unable to make enough insulin or use it properly to control blood glucose levels, diabetes develops. Increased chances of consequences including kidney failure, nerve damage, blindness, stroke, and heart attack are associated with diabetes. In diabetes management, the areas of focus include AI-powered wearable devices and mobile applications, AI-assisted insulin pumps, and personalized care. Figure 2 highlights the applications of AI in diabetes management.

### AI-powered mobile applications and wearable devices

#### AI-powered smartphone apps for diabetes management

The potential of AI can be harnessed to boost patient care, increase treatment effectiveness, and lowering healthcare costs. Continuous glucose monitoring, personalized treatment, and preventive measures are key to controlling diabetes^13^. AI-powered wearable devices and mobile applications are emerging technologies that hold promises for improving diabetes care by providing personalized feedback, analysis and personalized recommendations to patients based on real-time data collected from sensor or user inputs. Smartphone app ‘Dario’ is an AI-driven app that analyzes data from self-monitored blood glucose (SMBG) devices such as the Dario meter. Derozier *et al*.^14^ developed an AI algorithm for Android smartphones to cluster study data from glucometer data from patients into groups. For instance, AI-powered smartphone applications can also track insulin doses and blood glucose levels, and offer insulin injection recommendations as needed.

#### Remote management of GDM using AI apps

For remote management of GDM during the COVID-19 pandemic an AI-powered app, SineDie, was utilized in a study by Albert *et al*.^15^ SineDie analyzes the data pertaining to GDM and offers suggestions for dietary and insulin infusion. Despite the encouraging findings, certain limitations limit the generalizability of the findings of both studies that include small sample size, use of certain specific glucometer devices and lack of a control group.

#### Continuous glucose monitoring (CGM) and AI

Conventionally, invasive techniques are used for blood glucose measurements. Frequent finger pricking can be painful and inconvenient as well as may lead to exposure to various infectious antigens. Minimally invasive or noninvasive strategies would reduce the risks of infections and would be more practical for people with needle fear or gestational diabetes. Management of diabetes may require real-time monitoring of blood glucose levels by minimally invasive continuous glucose monitoring (CGM). Type 1 diabetes requires periodic insulin infusion, however, AI-powered CGM devices can calculate the insulin dosage required at a particular time period. Martina *et al*.^13^ utilized advanced tools such as AI-powered CGM for calculating insulin bolus. The study findings show that AI can be used for decision support systems especially for insulin-dependent diabetes to provide tailored suggestions regarding therapy modification.

#### Noninvasive blood glucose monitoring using wearables

AI-powered wearable devices can be used for noninvasive measurement of blood glucose levels. These devices alert users or caregivers when their blood glucose levels are abnormal. Pfützner *et al*.^16^ evaluated the accuracy of TensorTip Combo Glucometer (CoG), which has an invasive component using test strips and a noninvasive component to predict glucose levels from fingertip tissues. During a meal study, the noninvasive component showed acceptable accuracy as compared to the reference method used, that is, YSI 2300 Stat Plus. The study’s drawbacks include a small sample size, however, with further improvements to meet regulatory criteria, the device could enable frequent, reliable and painless glucose monitoring to improve medication adherence and patient outcomes in diabetic patients.

#### AI-assisted insulin pumps and artificial pancreas

AI and automated insulin delivery systems are transforming diabetes care. Insulin pumps are wearable devices, which help deliver specific doses of insulin at specific times. They are alternatives to multiple daily injections. Insulin pumps can help people with diabetes for management of insulin-dependent diabetes and lower the risks of complications. Recent studies have explored the efficacy of these AI-powered insulin pumps.

#### AI for insulin pump dosage calculation

Gómez *et al*.^17^ developed ensemble AI models based on neural networks, random forest, SVM, etc. to calculate basal insulin doses for type 1 diabetes patients using insulin infusion pumps. These models were tested on data from 56 patients using infusion pumps and continuous glucose monitors. The random forest-based model performed with higher accuracy in predicting the true basal insulin infusion rates.

#### Hybrid closed-loop artificial pancreas systems

Artificial pancreas, also known as closed-loop systems, automates insulin delivery through insulin pumps, CGM and AI algorithms. The MiniMed670G system is one of the first hybrid closed-loop artificial pancreas that automates the insulin dosage based on real-time glucose levels avoiding human error and reducing risks of hypoglycemia associated with insulin infusion. Forlenza *et al*.^18^ evaluated the hybrid closed-loop insulin delivery system in type 1 diabetes patients in a study. The study findings show improved glucose control especially in kids and youngsters aged 7 to 13 years with type 1 diabetes. Artificial pancreas holds promises for optimizing glycaemic control with reduced risks of hypoglycemia. The closed-loop system was compared to a sensor-augmented insulin pump by Brown *et al*.^19^ in a randomized multicenter trial over 6 months. The closed-loop system significantly enhanced the time spent in the controlled glycemic range regardless of patient’s baseline characteristics, demonstrating improved glycemic control. However, more adverse events of hyperglycemia occurred as a result of the failure of infusion pumps.

#### Adaptive closed-loop systems

Resalat *et al*.^20^ in a study proposed an adaptive model of predictive control for an artificial pancreas for type 1 diabetes management. Adaptive algorithms help reduce the artificial pancreas model-patient mismatch by individualized parameters such as insulin sensitivity. This model was adopted for postmeal insulin delivery based on prior glycemic patterns for the prevention of postmeal hypoglycemia. It also incorporated exercise metrics such as heart rate and accelerometry as inputs and demonstrated improved patient outcomes. The study included data from 99 type 1 diabetes patients and findings show adaptive approaches can significantly reduce hypoglycemia and rescue carbohydrates as compared to nonadaptive systems. However, to verify these advantages of adaptive closed-loop systems further clinical testing in real-world patients is required.

#### Comparative studies of closed-loop systems

Additionally, Paldus *et al*.^21^ compared the advanced Medtronic e-HCL (hybrid closed-loop) with the standard hybrid closed-loop system (s-HCL). The e-HCL study found that fewer alarms, and more time in a controlled glycemic range, showed advantages over the conventional system. However, the study’s drawbacks include a small sample size as only 11 persons participated in the trial and events such as ketoacidosis or severe hypoglycemia were not documented as in Brown *et al*.’s study. In summary, these studies provide insights into the potential of closed-loop systems and insulin pumps in enhancing glycemic control in insulin-dependent diabetes. integration of exercise and stress data could further optimize the performance of the models.

#### Reinforcement learning for insulin pumps

Some AI-assisted insulin pumps employ reinforcement learning to adapt their insulin infusion rate as per users’ behavior and preferences. Rigla *et al*.^22^ applied this decision support system in a pilot study on patients with GDM. Behavioral information gathered via wearables or mobile phones was used to investigate this AI approach to offer individualized lifestyle and counseling.

### AI-based patient outcomes predictions in diabetes

#### Treatment outcome prediction

Numerous researchers have evaluated the efficacy of AI-based diabetes technology in predicting the treatment outcomes and disease outcomes as well. In a study, Tarumi *et al*.^23^ applied the AI approach, Treatment Pathway Graph-based Estimation (TPGE), to predict treatment outcomes in type 2 diabetes mellitus patients using real-world electronic health record data. The integration of the model into the electronic health record (HER) system allows it to be accessed through a clinical decision support system (CDSS) web app. The CDSS predicts accurately the success rates of different medications for glycemic control targets. This system is designed to facilitate shared decision-making between clinicians and patients on optimal treatment strategies. However, further evaluation is required on the real-world impact of CDSS.

#### Digital diabetes prevention program evaluation

The AI-powered application named Lark DPP, a digital diabetes prevention program was evaluated in a study by Graham *et al*. in the prediabetic population^24^. The study included 3933 members data enrolled in Lark DPP. This study’s findings show that AI-powered DPP was highly effective when outcomes such as weight loss maintenance and peak weight loss were compared to in-person and other hybrid digital DPPs. The study suggests the application of AI coach in mitigating diabetes in a population at risk as a cost-effective way to reach more people at risk.

#### Diabetic foot ulcer risk prediction

AI can also predict the onset of diabetic foot ulcers in high-risk diabetes patients. An unsupervised learning technique was employed in a study^25^ to identify people at high-risk of developing foot ulcers with high accuracy by analyzing patient’s data and risk variables. The model achieved 90% accuracy with 100% specificity and 71% sensitivity demonstrating the feasibility of identifying risk variables in diabetic patients using AI that correlated with expert opinions.

#### Predicting hyper/hypoglycemia events

AI can also predict the events such as hyperglycemia and hypoglycemia in diabetes patients. Elhadd *et al*.^26^ employed an AI approach to predict hyperglycemia and hypoglycemia events during fasting during the holy month of Ramadhan in type 2 diabetes patients. Physical activity and current glucose levels on Ramadhan and non-Ramadhan days were used to train the XGBoost model which achieved higher accuracy in predicting normal glucose levels and hyperglycemic episodes but had limited ability to predict hypoglycemic episodes indicating the need for further model refinement.

#### AI-based insulin dose recommendations

AI algorithms can also suggest weekly insulin dosages. Lopez *et al*.^27^ utilized k-NN as a decision support system to identify the cause of hyperglycemia or hypoglycemia and adjust insulin dose accordingly. Upon validation on real-world human data, the AI system achieved an overall agreement of 67.9% with board-certified endocrinologists and delivered safe recommendations as per the endocrinologist’s review. The algorithm incorporates factors such as carbohydrates to insulin ratio for meal insulin doses, glucose trends, and smart bolus calculations.

#### Diabetic retinopathy screening

Preventing eyesight loss in diabetic patients is important as the main contributor to visual impairment is diabetic retinopathy, which significantly jeopardizes independence and quality of life. Prompt identification and treatment are the key to arrest the course of diabetic retinopathy and preserving the visual function. ML and DL are applied in diabetes to explore complications such as diabetic retinopathy. Various AI-based models have been developed that include EyeWisdom, EyeArt, CAREVL, etc. AI-powered EyeArt have been evaluated in studies by Lim *et al*.^28^ and Ipp *et al*.^29^

#### Performance of EyeArt AI system

Lim *et al*.^28^ compared the EyeArt AI system with general ophthalmologists and retina specialists for detecting diabetic retinopathy using 4-widefield stereoscopic dilated fundus photographs of 521 patients. The EyeArt system had much higher sensitivity for detecting more than mild diabetic retinopathy (mtmDR) than general ophthalmologists and retina specialists (0.96 vs. 0.27) but lower specificity (0.88 vs. 0.99). Moreover, the EyeArt AI system did not miss any case of vision-threatening diabetic retinopathy (vtDR) unlike few ophthalmologists. The false positive diagnosed by the AI system were mainly due to mild diabetic retinopathy or some other underlying pathology. However, the study findings suggest the EyeArt AI system could be an essential screening tool for diabetic retinopathy as it may help address the screening burden.

#### Evaluation of EyeArt for undilated retinal imaging

Ipp *et al*.^29^ evaluated the EyeArt AI system for diagnosis of mtmDR and vtDR using undilated 2-field retinal images of 893 participants while taking 4-field stereoscope dilated images as reference standard. The AI system demonstrated higher sensitivity and specificity for detecting mtmDR (sensitivity 0.95 and specificity 0.87) and vtDR (sensitivity 0.97 and specificity 0.90) as compared to the reference standard. Ipp *et al*.’s study findings suggest that EyeArt AI system can accurately and safely detect mtmDR and vtDR without the need for dilation. This could further facilitate screening in primary care settings and improve adherence to screening guidelines as well.

#### EyeWisdom AI system for retinopathy screening

Another AI-based screening system EyeWisdom was evaluated in a study by Pei *et al*.^30^ that included 549 T2DM patients. Using a nonmydriatic fundus camera and cloud-based image processing software, researchers performed the fundus inspection. The AI-based EyeWisdom system could diagnose and identify various stages of diabetic retinopathy.

#### CAREVL AI model for retinopathy detection

Channa *et al*.^31^ developed CAREVL to compare AI and ophthalmologists’ detection of diabetic retinopathy. The model estimated that at 5 years, AI screening would result in less vision loss compared to ophthalmologists’ screening due to higher patient acceptance of screening and treatment compliance. However, the performance of these AI-based screening models can be enhanced further by modifiable variables such as treatment adherence.

#### Identifying hospitalization risk individuals in diabetes

AI plays a critical role in predicting hospitalization risks in diabetes patients. Once these high-risk individuals are identified, healthcare professionals can take proactive measures to prevent hospitalization. Lo-Cigani *et al*.^32^ used a random survival forest to identify significant predictors for hospitalization in type 2 diabetes patients. After training the algorithm on diabetes type 2 Medicaid enrollees’ data, a survival tree was set to identify adherence thresholds to oral hypoglycemics. The study found that the commonly used 80% adherence threshold was not optimal for predicting hospitalization risks among all patient subgroups. The most discriminating risk of hospitalization was nonuniform and varied from 46 to 94% depending on the patient’s health and medication complexity. However, the study found ML approaches intuitive and appropriate to determine patient’s specific adherence threshold.

### Identifying disease markers

#### Demographic risk factors using ANN Model

Large-scale datasets can be analyzed using AI to identify disease markers. In Borzousei’s study^33^, a multilayer ANN model was developed on a sample of 234 individuals to identify important demographic risk factors for type 2 diabetes. Among 234 individuals, only 151 were diabetics already diagnosed with HbA1c criteria. The model identifies waist circumference, age, BMI, hypertension, stress, smoking, and family history of type 2 diabetes as predictors. Among these predictors waist circumference and age were the most important predictors. Therefore, the study suggests these easily measurable factors can be helpful for type 2 diabetes risk assessment and screening tests, especially in low resource areas compared to medical variables that require laboratory tests.

#### Machine learning for novel biomarker identification

Hathaway *et al*.^34^ and others by using machine learning, were able to identify novel as well as the most relevant biomarkers associated with type 2 diabetes mellitus by integrating physiological, biochemical, and sequencing datasets. They used physiological, biochemical, and sequencing data for each patient, and machine learning was applied using SHapley Additive explanations (SHAP). This allowed for binary (no diabetes or type 2 diabetes) and multiple classifications (no diabetes, prediabetes, and type 2 diabetes) of the patient group, both with and without the inclusion of HbA1c levels. The results were verified using various models, including logistic regression (LR), linear discriminant analysis (LDA), Gaussian Naïve Bayes (NB), support vector machine (SVM), and Classification and Regression Tree (CART), with 10-fold cross-validation.

#### Refining biomarker accuracy with machine learning

By exploring previously published metadata sets, Kavakiotis *et al*.^35^ applied machine learning in refining the accuracy of biomarkers used to characterize the pathology as well as to highlight vulnerable populations in need of clinical intervention.

#### Integrating biomarkers for improved prediction

In a study by Jelinek *et al*.^36^, machine learning technique revealed that coupling HbA1c with additional biomarkers, such as 8-hydroxy2-deoxyguanosine (8-OhdG) and other metabolites, can increase the accuracy of the predictive model and better characterize the severity of the disease. The study utilized machine learning to predict type 2 diabetes status by integrating cardiac physiological, biochemical, genomic, and epigenomic biomarker data in a patient-matched manner. In their study, 50 patients were analyzed, and the results showed that machine learning algorithms could identify the interconnectedness between diabetic classification, mitochondrial function, and methylation status. The research demonstrated the potential of novel biomarkers to complement existing diagnostic standards and provide more precise methods for identifying the development and severity of type 2 diabetes mellitus in at-risk populations, such as those with prediabetes. The study aimed to determine the best predictive features and whether they could be used alone or in combination with HbA1c. Although some models did not achieve predictive accuracy above 50%, their inclusion was intended to contrast them against the models that did rise above 50% without HbA1c and identify the best overall predictors among the biomarkers.

### Application of disease markers to improve performance of predictive models

#### Improving type 2 diabetes risk prediction with polygenic risk scores and metabolites

In a population-based 10-year prospective cohort study, Hahn *et al*.^37^ applied machine learning to the prediction of type 2 diabetes using genome-wide polygenic risk scores and metabolic profiles in the Asian population. The study revealed that the addition of serum metabolites to the risk prediction model for type 2 diabetes, in conjunction with clinical and genetic factors, can moderately enhance its accuracy. The research team compared a random forest-based machine learning model with a standard binary logistic regression model and found that the former was more effective in improving predictive power, including discrimination performance and reclassification improvement. The machine learning model was also found to be comparable to the logistic regression model in terms of interpretability.

The study used four different sets of independent variables (demographic, medical history, clinical, genetic, and selected metabolites) with logistic regression (LR) and random forest (RF) algorithms to predict incident type 2 diabetes. The models were evaluated using AUC, Brier score, and log-loss metrics, as well as NRI, cNRI, and IDI indices. This study found that adding a genetic risk score (gPRS) to traditional clinical risk factors modestly improved the accuracy of predicting type 2 diabetes risk. Previous studies on PRS-based type 2 diabetes risk prediction models in Asian countries did not consistently outperform conventional risk models, especially when glucose or HbA1c were included as predictors. However, gPRS, which captures an individual’s comprehensive genetic predisposition for type 2 diabetes, improved the model’s performance even in the presence of glucose and HbA1c as clinical factors. These findings were consistent with Khera *et al*.’s study^38^ showing that including gPRS significantly improved the type 2 diabetes risk prediction ability in terms of AUC.

## Limitations and future outlook

### Current challenges of AI in diabetes care

#### Data limitations

More than one-third of the studies have highlighted the data-related challenges as significant hurdles in leveraging AI for diagnosis and management. Insufficient data, biased or skewed datasets, and varying quality of medical images can lead to poor generalizability, algorithm biases, and inaccurate diagnosis^39^. Errors in data labeling also hinders the models’ ability to make reliable predictions. Moreover, AI models lack the ability to grade severity of various diabetes complications such as diabetic retinopathy and diabetic foot ulcers, which is crucial for clinical decision-making and treatment^40^.

#### Clinical integration challenges

Although AI has made significant progress, AI algorithms still need further refinement for accurate predictions across diverse clinical scenarios. Translating the promising performance of AI models from controlled research settings to the multifaceted real-world clinical environments is a major concern for developers. The integration of AI in existing clinical workflows is essential for widespread adoption of AI-devices. Moreover, the development of transparent and interpretable models is crucial for building trust among healthcare professionals and patients who currently regard AI as the ‘black box’^41^. With development of interpretable models and clinicians’ training and patients’ acceptance would play key role in smooth integration of AI in clinical settings.

### Future directions of AI in diabetes care

#### Data enhancements

There is a need to overcome data limitations. The collaborations of multiple organizations can help create large, diverse datasets essential for AI models’ training. Acquisition of high-quality images and diverse data that covers various demographic and clinical aspects is vital to improve model generalizability^39^.

#### Multimodal AI models

AI models, that not only provide predictions but also describe the underlying pathological process, are crucial for building clinician and patient trust. Multimodal AI models that incorporate divers data types like imaging, genomics, and clinical presentations can enhance the diagnostic accuracy of diabetic complications and their severity, enabling personalized treatment.

#### Interdisciplinary approach

Future research should prioritize interdisciplinary collaboration between computer scientists, endocrinologists, data analysts, and patient advocacy groups. This approach can lead to AI-based diabetes solutions that are not only technically advanced but also clinically relevant and patient-centered. Such collaborations could result in more accurate diagnostic tools, personalized treatment plans, and improved patient outcomes, while addressing ethical considerations and diverse patient needs

#### Clinical validation and patient-centric AI applications

The real-world implementation and continuous monitoring of these AI models in clinical environments are crucial to validate their effectiveness, reliability, and integration into clinical workflows. With the passage of time due to ongoing research, AI technologies are evolving and incorporation of feedbacks from practical applications can be utilized to refine and improve AI models’ performance. Patient centric AI applications should be prioritized, empowering patients through portable devices that facilitate self-management and early screening of potential complications.

## Ethical approval

Not applicable.

## Consent

Not applicable.

## Source of funding

Not applicable.

## Author contribution

M.I.: conceptualization, methodology, data curation, formal analysis, writing of initial and final draft, visualization, and supervision; M.S.: methodology, validation, data curation, and writing initial and final draft; S.N.Q.: data curation, writing initial and final draft, and visualization; R.A.: methodology, reviewing and editing, data curation, and formal analysis; H.M.: data curation, writing initial draft, and visualization; M.R.: supervision, editing and reviewing, and writing final draft; I.U.: writing final draft, formal analysis, visualization, and funding acquisition; I.-u.-d.: data curation, formal analysis, visualization, and methodology; S.N., Z.E., and M.K.: writing final draft, editing, and reviewing; E.R.: methodology, validation, and data curation. All authors approved the final manuscript.

## Conflicts of interest disclosure

The authors declare that they have no financial conflict of interest with regard to the content of this report.

## Research registration unique identifying number (UIN)

1. Name of the registry: not applicable.
2. Unique identifying number or registration ID: not applicable.
3. Hyperlink to your specific registration (must be publicly accessible and will be checked): not applicable.

## Guarantor

Sardar Noman Qayyum, MBBS; Department of Internal Medicine, Bacha Khan Medical College, Mardan, Pakistan. E-mail: dr.sardarnoman@gmail.com; https://orcid.org/0009-0005-9132-7256.

## Data availability statement

Not applicable.

## Provenance and peer review

Not commissioned, externally peer-reviewed.
